# Molecular epidemiology of the HIV-1 epidemic in Fiji

**DOI:** 10.1038/s44298-024-00019-3

**Published:** 2024-03-06

**Authors:** Atlesh Sudhakar, Donald Wilson, Rachel Devi, Dashika Anshu Balak, Jenni Singh, Kesaia Tuidraki, Lavenia Gaunavinaka, Waisale Turuva, Taina Naivalu, Blair Lawley, John H. Tay, Francesca Di Giallonardo, Sebastian Duchene, Jemma L. Geoghegan

**Affiliations:** 1 Fiji Institute of Pacific Health Research, Fiji National University, Suva, Fiji; 2 Department of Microbiology and Immunology, University of Otago, Dunedin, 9016 New Zealand; 3 Ministry of Health, Suva, Fiji; 4 Sexual and Reproductive Health Clinic (Central Eastern Division), Suva, Fiji; 5 Reproductive Health Clinic, Suva Hub, Fiji; 6 Medical Services Pacific (MSP), Suva, Fiji; 7 Reproductive Health Clinic, Western Hub, Suva, Fiji; 8 Reproductive Health Clinic, Northern Hub, Suva, Fiji; 9 Department of Microbiology and Immunology, Peter Doherty Institute for Infection and Immunity, University of Melbourne, Melbourne, VIC Australia; 10 The Kirby Institute, University of New South Wales, Sydney, 2052 NSW Australia; 11 Department of Computational Biology, Institut Pasteur, 75015 Paris, France; 12 Institute of Environmental Science and Research, Wellington, 5018 New Zealand

**Keywords:** Computational biology and bioinformatics, Genetics

## Abstract

Very little is known about the HIV-1 epidemic in Fiji, nor the wider South Pacific region more generally, yet new reported HIV-1 infections are on the rise. As of 2023, there are an estimated 2000 cases of HIV-1 in Fiji with heterosexual contact the primary route of transmission. In this study, we used a molecular epidemiological approach to better understand the genetic diversity of the HIV-1 epidemic in Fiji and reveal patterns of viral transmission. Between 2020 and 2021, venous blood samples were collected from people who had previously been diagnosed with HIV-1. We generated molecular data from 53 infections, representing ~2–3% of reported cases, to identify HIV-1 subtypes and determine the outbreak’s trajectory. Among the 53 HIV-1 cases, we used Bayesian inference to estimate six separate introductions with at least two of these introductions leading to sustained transmission forming large, nation-wide clusters of HIV-1 subtype C. We found that since the introduction of public health interventions circa 2014, the effective reproductive number, *R*_*e*_, decreased among the major clusters identified from an average of 2.4 to just below 1. Molecular epidemiological analysis suggested that public health efforts aimed at decreasing the spread of the disease were at least somewhat effective. Nevertheless, with a recent rise in reported HIV-1 cases, this study demonstrates the utility of molecular data to inform a more targeted public health approach for controlling its spread.

## Introduction

Fiji, an archipelago of over 330 islands situated in the southwest Pacific, is the second-highest country in the Asia-Pacific region for new HIV-1 infections^1^. There are currently over 2000 known cases of HIV-1 and AIDS in Fiji^2^, of which only approximately 40% have access to antiretroviral therapy (ART). Since the first reported case in 1989, HIV-1 incidence in Fiji has increased by about ten-fold, from 0.7 per 100,000 in 2000 to 7 per 100,000 in 2021. While this increase is largely due to improved diagnostic capacity, it is also suspected to reflect a growing epidemic. Consequently, since 2014 there has been a national effort to reduce HIV-1 transmission in Fiji using contact tracing, educational campaigns, increasing treatment accessibility, improving counseling services and decentralising testing^3^. Nevertheless, the high prevalence of sexually transmitted infections more generally is suggestive of a large at-risk population^4^.

At a molecular level, there is very little known about the Fijian HIV-1 epidemic. A previous subtyping effort of just 27 patient samples revealed that HIV-1 cases in Fiji were largely caused by subtypes B and C, with multiple introductions into the country^5^. Within the wider region, Oceania has low absolute case numbers of HIV-1 where epidemics in Australia are dominated by subtype B, with a smaller proportion of subtype C and the recombinant CRF01_AE^6^. Among Pacific Island nations, however, the genetic diversity of HIV-1 infections is not well understood due to under-resourced surveillance systems for monitoring such infections. Due to this under-resourcing, it is likely that HIV-1 incidence rates in Pacific Island nations are much higher than currently reported.

Inferring HIV-1 transmission dynamics in Fiji relies on basic patient demographic data to inform contact tracing. However, by combining molecular data with individual-level meta data, we can more accurately estimate parameters of such viral outbreaks. These tools can estimate: the effective reproductive number (*R*_*e*_) – extending to *R*_*e*_ for individual viral lineages; the effective population size which reflects the number of infected individuals; and the number of introductions of a pathogen into the country. In addition to providing essential information on the pattern of viral evolution and epidemiology during epidemics, viral genomic data has direct public health importance^7^. When combined with geographic and population information, these data can reveal pathways of viral spread that can be used to create effective interventions. Temporal and geographic information can be integrated into phylodynamic analyses to detect the spread of the pathogen in time and space^8^, develop predictions about an outbreak’s trajectory^9^, and identify clusters of similar sequences as an indication of possible transmission hotspots^10^. Herein, we generated HIV-1 molecular data from individuals in Fiji to investigate virus introductions, the extent and pattern of viral spread and the effectiveness of public health measures used to curb the spread of the disease.

## Materials and methods

### Ethics

The study was approved by the College Human Health Research and Ethics Committee (CHHREC), Fiji National University, Ministry of Health and Medical Services (MoHMS) of Fiji and the University of Otago Human Ethics Committee. Written informed consent was obtained from the participants to participate in the study.

### Sample collection, PCR and genetic sequencing

Blood samples were collected from people living with HIV-1 who attended their routine clinic visits at the three facilities around Fiji (Northern, Western and Central Reproductive Health Clinics) over a 5-month period from November 2020 to March 2021. A total of 222 samples were collected from both antiretroviral-naïve and antiretroviral-experienced HIV-1-positive Fijian individuals. RNA was extracted and purified using the Qiagen QIAamp Viral RNA Isolation kit in Fiji. As part of a different study aimed at detecting HIV-1 drug resistance, RNA was purified and three coding regions of HIV-1 were amplified using QuantaBio qScriptTM® Flex cDNA Synthesis Kit. Each fragment corresponded to gag-p2/NCp7/p1/p6/, pol-PR/RT-(1657 bp fragment), pol-IN-(1114 bp fragment), and env-C2V3- (480 bp fragment) (see Supplementary Table 1 for primer details). Out of the 222 samples, 57 tested positive upon PCR for all three target genes and were subject to genetic sequencing. The amplified positive PCR products were cleaned using the QIAquickR PCR Purification Kit (Qiagen). The Nextera sample preparation kit (Illumina) was used to prepare libraries that were sequenced using the Illumina MiSeq platform.

### HIV polymerase gene assembly

We focused assembly on the polymerase (*pol*) gene since it is appropriately conserved to infer phylodynamic parameters. Raw sequencing reads were first assembled de novo using Trinity^11^ and contigs were subject to a sequence similarity search using BLAST against a custom HIV *pol* gene database. Contigs that were annotated as an HIV-1 *pol* gene were further subject to a BLAST search using NCBI’s entire nucleotide database to obtain the closest known genetic match for each, which was subsequently used as a reference to map all pol gene contigs using bowtie2^12^ for each sample. The subtype of assembled pol genes was estimated using the REGA HIV-1 subtyping tool v3^13^. In total, 53 HIV-1 pol genes were successfully assembled with >50% non-ambiguous nucleotides.

### HIV phylogenetics

We selected a random sample of 2000 publicly available *pol* gene sequences from the Los Alamos National Laboratory HIV database (LANL) (https://www.hiv.lanl.gov) that fell across subtypes B and C to represent a global sample. We selected a further 500 *pol* sequences from GenBank that were the closest known genetic relatives of the *pol* gene sequences generated here using a BLAST search. These sequences, that included sampling location and year, were aligned with the 53 Fijian *pol* gene sequences using MAFFT^14^ and a maximum likelihood phylogenetic tree was estimated using IQ-TREE^15^ using the HKY + G substitution model^16^. Sequences containing >50% ambiguous regions or missing year of sampling were removed, resulting in a total of 2505 sequences. We regressed root-to-tip genetic divergence against sampling dates (year) to investigate the evolutionary tempo of this data set using TempEst^17^. See Supplementary Table 2 for a list of accessions for global data used.

### Discrete phylogeography and inference of importation events

Of the data set described above, 1308 *pol* gene sequences included the exact date (day) of sampling, including the 53 from Fiji generated in this study. Using this refined data set, we estimated a phylogenetic tree under maximum likelihood using IQ-TREE v2^18^ and the HKY + G substitution model. To infer an evolutionary timescale, we fit a molecular clock as implemented in LSDv0.3^19^ with the sampling times for calibration and a fixed substitution rate of 3 × 10^−3^ subs/site/year, like that estimated by Faria^20^.

To infer the number of introductions we treated the geographic location as a discrete trait, with Fijian samples assigned to Fiji, and those from other locations to their corresponding continents. Under this method, discrete phylogeography^21^, the migration process is a Markov chain with geographic locations corresponding to the states of the chain and their migration routes selected using Bayesian stochastic search variable selection^22^. We specified this model in the Bayesian phylogenetics platform BEASTv1.10^23^. Due to the size of our data set, we chose to fix the phylogenetic tree and its corresponding branching times, as estimated above. We sampled the posterior distribution using Markov chain Monte Carlo (MCMC) with a chain length of 10^7^, sampling every 10^3^ steps. We determined sufficient sampling by verifying that the effective size of all parameters was at least 500 using Beastiary v1^24^.

### Estimates of epidemiological parameters using phylodynamics

We analysed the two major transmission clusters under a birth-death skyline model^25^, as implemented in BEASTv2.5^26^. Because we were interested in understanding the broad epidemiological dynamics, we analysed them both under a single model. The benefit here is that we can leverage information from both clusters to inform shared parameters, such that they correspond to weighted averages. This approach has demonstrated good performance in analyses of multiple transmission clusters^27^. We fixed the substitution rate to 3 × 10^−3^ subs/site/year and specified the HKY + G substitution model. Our choice for this substitution model, over those that involve invariant sites follows its trade-off between biological interpretability and statistical complexity^28^.

Each cluster had an independent time of origin, but the remaining parameters of the birth-death skyline model were shared between them. The effective reproductive number, *R*_*e*_, was sliced over four time periods; from the time of origin until 2015, from 2015 to 2017, from 2017 to 2019, and from 2019 to the date of the last collected sample. This model requires strong prior knowledge of the sampling proportion or the time over which an individual is infectious (i.e. not on treatment), *D*, which is sometimes referred to as the ‘duration of infection’ and is equivalent to the inverse of the becoming uninfectious rate. We expect *D* to be impacted by public health interventions, such as contact tracing and the use of antiretroviral therapy, which likely reduces the time over which an individual can effectively infect others. We set *D* to have a fixed value of five years prior to 2014, because this is the year when contact tracing was effectively introduced in Fiji^3^. From 2014 to the date of the last collected sample in 2021, we set a shorter *D* with two possible values, *D* = 1 year, or *D* = 6 months. These analyses allow us to assess the robustness of *R*_*e*_ to different values of *D* and reflected our uncertainty about the time from infection to starting treatment and thus becoming uninfectious^29^.

Because *D* is fixed in our analysis, the remaining parameters require prior probability distributions. For *R*_*e*_ we used a lognormal prior with mean of 0 and standard deviation of one. For the sampling proportion we used a Beta distribution with parameters alpha and beta = 1, such that it is flat across its domain. To sample the posterior distribution, we used MCMC with a chain length of 10^7^, sampling every 10^3^ steps and verified sufficient sampling as in our phylogeographic analyses above.

## Results

### Demographics of HIV-1 cases sequenced in Fiji

**Fig. 1:**
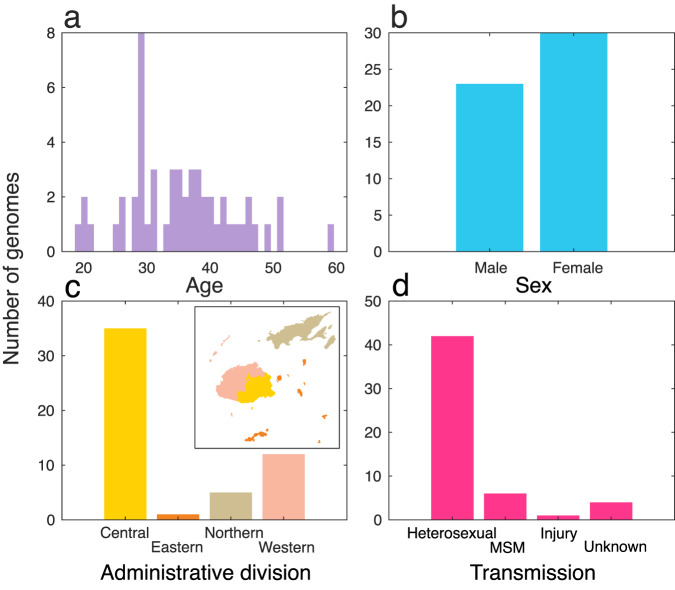
Patient demographics and geography for HIV-1 genetic data generated in this study. **a** Age; **b** sex; **c** administrative division of testing laboratory, including an insert map of Fiji with corresponding colours; and **d** reported mode of transmission.

We generated genetic data from 53 people with HIV-1 in Fiji collected between 2020 and 2021. Of these HIV-1 cases, 44% were male and 56% were female, and the median patient age was 35 (Fig. 1). The vast majority (79%) were attributed to heterosexual transmission while 11% were among men who have sex with men (MSM). Most genetic data were generated from HIV-1 infections reported in the Central administrative division, reflecting the most populous division in Fiji.

### Genetic diversity and identification of major HIV-1 clusters in Fiji

**Fig. 2:**
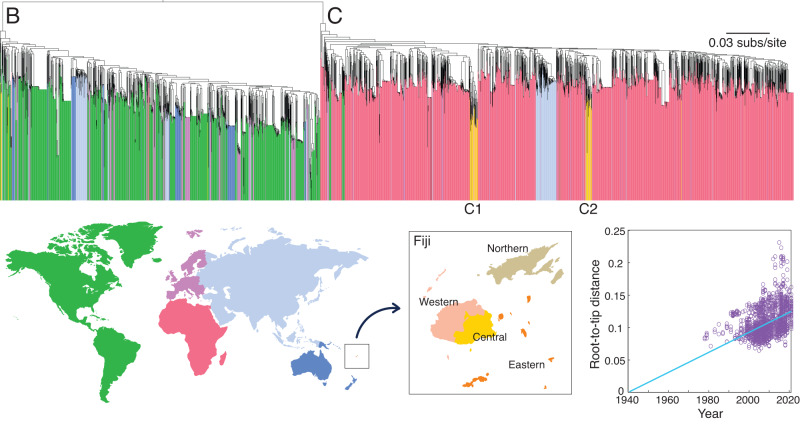
Phylogeography and genetic diversity of HIV-1 in Fiji. Maximum likelihood phylogenetic tree of HIV-1 pol genes across subtypes B and C from Fiji as well as a random global sample. Colours in the map denote the sampling location for each sequence. The two major clusters identified in Fiji are labelled C1 and C2. Branch lengths depict the number of nucleotide substitutions per site. A root-to-tip regression versus sampling date for each sample within the phylogeny is shown, where Pearson’s correlation coefficient = 0.43 (*p* < 0.001).

HIV-1 sequences generated here fell across subtypes B and C, with the majority (87%) identified as the latter (Fig. 2). The reconstructed maximum likelihood phylogenetic tree shows many of the sequences grouped within two major Fijian HIV-1 clusters, indicative of sustained transmission within Fiji. These clusters comprised 24 and 19 genomes each, representing 81% of Fijian HIV-1 sequences generated here. Both Fijian clusters were identified as subtype C with the closest known genetic relatives sampled in Africa. Other Fijian sequences fell within clusters containing between one and five HIV-1 genomes across both subtypes. In addition, we inferred a significant temporal signal in the genetic divergence of these data overall (Fig. 2).

**Fig. 3:**
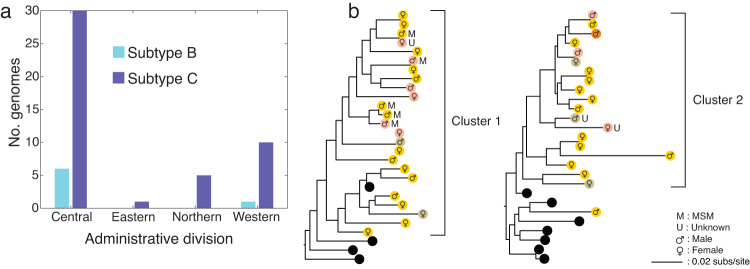
Geography of HIV-1 subtypes in Fiji. **a** Number of pol gene sequences falling across subtypes B (light blue) and C (dark blue) from each administrative division. **b** The two major clusters (more than four sequences) identified in Fiji from those samples, colour-coded by sampling location (i.e. administrative division) shown on the map in Figs. 1 and 2 (black circles indicate global pol genes). The mode of transmission for MSM (M) and unknown (U) is shown while all others are due to heterosexual transmission. Patient sex (male or female) is also shown. Branch lengths indicate the number of nucleotide substitutions per site.

While subtype B sequences were only present in the Western and Central administrative divisions, encompassing the main island of Viti Levu, subtype C sequences were present in all four administrative divisions (Fig. 3). We found that five out of six cases among MSM fell within one of the large Fijian clusters (Cluster 1). The two clusters contained numerous small clades of two or three sequences with high genetic similarity. Cluster 1 contained a clade of three sequences from infections among MSM and another three sequences among individuals reporting heterosexual risk exposure. Notably, in the latter case, all three infections were from different geographic areas. The relatively high number of genetically similar pol gene sequences from females reporting heterosexual transmission suggested unsampled infections among males. Overall, cases from all administrative divisions were mixed without any evidence of geographic clustering (Fig. 3).

### HIV-1 phylogeography in Fiji using Bayesian inference

Our discrete phylogeographic analyses estimated the number of migration events from international locations required to explain the data at hand (1308 international samples and 53 from Fiji). The number of migration events from any geographic location into Fiji was six (maximum *a posteriori* estimate, with a posterior probability of 0.96). Most of these migration events had a putative origin in Africa (maximum *a posteriori* = 4 events, posterior probability = 0.93) or North America (maximum *a posteriori* = 2, posterior probability = 0.94), with no migration events inferred from Asia or Europe. Crucially, however, our inference of the locations of origin are limited by the fact the data set is heavily biased towards samples from Africa and North America.

We inferred the approximate date of importation events by identifying the corresponding branches in the phylogenetic tree. Importations could have occurred at any point in time between the stem and the crown nodes. Stem nodes had dates between 1980 and 2002 and the crown nodes between 1996 and 2017. The very wide uncertainty likely reflects sparse sampling of the lineages leading up to importations into Fiji.

### Bayesian phylodynamic inference of epidemiological parameters for HIV-1 in Fiji

The posterior trajectory of *R*_*e*_ indicated that most of the spread in these data likely occurred before 2017, regardless of our assumption of *D*. We report the weighted average for the main two clusters and assuming *D* = 1 year prior to 2014. The first time-interval, before 2015, had a posterior *R*_*e*_ mean estimate of 2.4 (95% credible interval: 1.88–2.90). The date of origin of this interval was around 2001 for both transmission clusters (for the first cluster, 95% credible interval from early 2001 to mid-2003 and from mid-2001 to early 2004 for the second cluster).

**Fig. 4:**
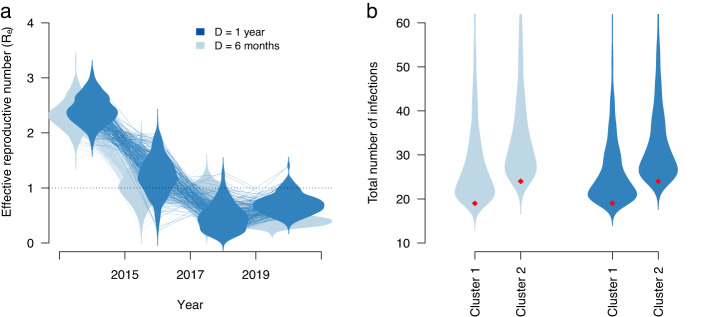
Epidemiological parameters inferred using Bayesian phylodynamic analyses of the two largest Fijian clusters identified with these samples. **a** Posterior distribution of the effective reproductive number, *R*_*e*_, across four intervals of time (up to 2015, from 2015 to 2017, from 2017 to 2019, and from 2019 to the date of the last collected sample, 2021). The dark blue corresponds to the assumption of a duration of infection, *D*, of 1 year and the light shade indicates *D* of 6 months after 2014. The violins denote the posterior density, and the lines represent the trajectory sampled from the posterior. **b** Posterior distribution of the total number of infections in each Fijian cluster under the two possible values of *D*. The red diamonds denote the number of sequences in the data set.

In the second interval, between 2015 and 2017, the mean posterior *R*_*e*_ was 1.2, but with an uncertainty that included a value of one (i.e. no epidemic spread; 95% credible interval: 0.54–1.90), such that evidence for spread was less strong. In the subsequent intervals, between 2017 and 2019, and thereafter, the posterior density of *R*_*e*_ was concentrated below 1.0, with a mean of 0.47 (95% credible interval: 0.07–0.93) and 0.71 (95% credible interval: 0.38–1.06), respectively (Fig. 4). Because *R*_*e*_ is expected to scale positively with *D*^30,31^, we conducted a sensitivity analysis in which we set *D* = 1 year for the entire duration of the outbreak clusters. Under this assumption, the absolute *R*_*e*_ values for the first two intervals were lower (means of 1.26 and 1.45), but we observed the same trend, where *R*_*e*_ decreased below 1.0 only after 2017.

Our phylodynamic analyses also provided an estimate of the total number of infections in each transmission cluster since the first sequenced case, which we calculated by taking the number of samples in each cluster divided by the sampling proportion parameter. Again, both assumptions about *D* resulted in similar posterior distributions. The estimated numbers of total infections had means of 23.62 (95% credible interval: 19.14–50.66) and 29.84 (95% credible interval: 24.19–63.99). These values correspond to a very high sampling proportion with a mean of 0.80 (95% credible interval: 0.38–0.99). Thus, the sequence data here represent most of the total number of infections within these clusters.

## Discussion

Fiji has the second-fastest growing HIV-1 epidemic in the Asia-Pacific region, yet little is known about the genetic diversity that shapes this outbreak, nor the effect of public health interventions on its trajectory. In this study, we generated 53 HIV-1 pol gene sequences to better understand the HIV-1 outbreak in Fiji. Although these data represented only ~2–3% of the country’s reported HIV-1 cases, it is the most HIV-1 genetic data produced from any small island country in the South Pacific to date. Our molecular epidemiological analysis of HIV-1 in Fiji identified two large transmission clusters containing sequences within subtype C. While both subtypes B and C were identified, we found that only subtype C was associated with substantial locally sustained transmission. Our epidemiological parameter estimates therefore apply to the genetic diversity captured here and not that of potential undetected transmission clusters.

Among the genetic data from 53 HIV-1 samples, we inferred six separate introductions of the virus into Fiji, where at least two of these introductions led to sustained transmission. Within the two large clusters identified, ongoing transmission was likely sustained for approximately 20 years followed by a dramatic decrease circa 2014. Indeed, 2014 coincided with an increase in public health interventions in Fiji such as contact tracing, educational campaigns and increasing treatment and counseling accessibility^3^. While concerns remain as to the growing HIV-1 outbreak, our phylodynamic analysis estimated that the effective reproductive number (*R*_*e*_) fell from a mean of 2.4 before 2015 to less than 1.0 after 2017, suggesting that public health efforts aimed at decreasing the spread of the disease were at least somewhat effective. Moreover, our estimates of the epidemic size of the two largest clusters were indicative of a very high sampling proportion of these outbreaks, meaning that contact-tracing efforts likely identified and sequenced most cases in these clusters. However, it is important to note that cases which are not sequenced here are likely to consist of both unsampled transmission clusters and those with international links.

It was noteworthy that five out of the six cases associated with MSM were found among a single cluster comprising 24 Fijian cases. Although only three sequences from MSM-associated infections formed a genetic cluster in the phylogenetic tree, it is plausible that there is a larger non-disclosed or unsampled MSM-associated group of HIV-positive individuals driving this transmission chain^32^. Nevertheless, the ~1.6:1 male-to-female ratio among heterosexual and unknown transmission within the cluster suggests heterosexual transmission remains the main exposure risk. Future work should focus on assessing the extent to which risk groups contribute to overall spread^31^.

The geographic origin of the large transmission clusters detected here suggested a genetic link to Africa where subtype C is most commonly sampled^33^. The genetic diversity of HIV-1 is highest in Africa and there are obvious geographic blind spots with little or no molecular data^34–36^, and thus the genetic link does not imply a geographic origin for these introductions. Within Fiji, we found no geographic effect on transmission where sequences from the Central administrative division were sampled more frequently, likely reflecting the increased testing capacity of the region compared with outer, more rural islands.

This study has shown that an HIV-1 molecular epidemiological surveillance system in Fiji could provide, in real-time, a better understanding of the HIV-1 transmission dynamics particularly among high-risk populations. Indeed, such data can inform contact tracing, detect new introductions, and identify expanding transmission clusters. Our results are the first to show how both new introductions as well as sustained transmission has largely driven the epidemic in Fiji. We conclude that public health efforts have likely decreased the rate of transmission over the last decade of the clusters identified here. Nevertheless, with a recent rise in reported HIV-1 cases, this study demonstrates the utility of molecular data to inform a more targeted approach for public health.

## Supplementary information

Supplementary Information

## Data Availability

Raw sequencing reads are available within NCBI’s SRA under BioProject PRJNA1021670. The 53 *pol* gene sequences are available under GenBank accessions OR621095-OR621147.
