# Supplementary material for: Molecular epidemiology of the HIV-1 epidemic in Fiji
Source: Npj Viruses. 2024 Mar 6;2:8. doi: 10.1038/s44298-024-00019-3 (PMC11721668; doi:10.1038/s44298-024-00019-3)
Supplement: Supplementary file 1 — Supplementary Information [file 44298_2024_19_MOESM1_ESM.pdf]

Supplementary Table 1. Primers used for amplification of three fragments as well as for cDNA.

Primers for cDNA			
**#**	**Primer Name**	**Sequence (5'-3')**	**Designated Fragment**
1	Half RT	TATTTCTGCTATTAAGTCTTTTGATGGGTCA	Fragment 1
2	Ext B int 11	TATGTTGTTATTACTAATTTAGCATCCCCTARTGGDATRTGTACTTCYGA	Fragment 3
3	ED 12	AGTGCTTCCTGCTGCTCCCAAGAACCCAAG	Fragment 4

Primers for Primary External PCR			
**#**	**Primer Name**	**Sequence (5'-3')**	**Designated Fragment**
1	Ext F p7.9(forward)<br>Half RT (reverse)	AAAGCATTGGGACCAGGAGCGACACTAGAAGARATGATGACAGCATGYCA<br>TATTTCTGCTATTAAGTCTTTTGATGGGTCA	Fragment 1
2	F int 9 (forward)	AGTTAATAAAAAAGGAAAAAGTCTACCTGGCATGGGTNCCRGCACAYAAR	Fragment 3

	Ext B int 11 (reverse)	TATGTTGTTATTACTAATTTAGCATCCCCTARTGGDATRTGTACTTCYGA	
3	ED5 (forward)<br>ED105 (reverse)	ATGGGATCAAAGCCTAAAGCCATGTG<br>GCTTTTCCTACTTCCTGCCAC	Fragment 4

**Primers for Secondary Nested PCR**			
**#**	**Primer Name**	**Sequence (5'-3')**	**Designated Fragment**
1	Ext p7.10 (forward)<br>Half Pol (reverse)	GGAGCGACACTAGAAGAAATGATGACAGCATGYCARGGAGTRGGRGGRCY<br>TCTGCCAGTTCTAGCTCTGCTT	Fragment 1
2	F int 8 (forward)<br>Ext B int 12 (reverse)	TGGCATGGGTACCAGCACACAAAGGAATTGGRGGRAATGARCARGTRGAY<br>ACTAATTTAGCATCCCCTAGTGGGATGTGTACYTCTGARCTTAYTYTTGG	Fragment 3
3	E80 (forward)<br>ED 125 (reverse)	CCAATTCCCATACATTATTGTG<br>CAATTTCTGGGTCCCCTCCTGAGG	Fragment 4

Supplementary Table 2. A list of GenBank accession numbers for the global genomes used in this study.

GenBank accession	Sampling region
AF110977	Africa
KY658711	Africa
KY658704	Africa
AF443109	Africa
AF443077	Africa
AF443088	Africa
AF110972	Africa
KY658710	Africa
AF110965	Africa
AF443076	Africa
AF443079	Africa
AF443104	Africa
MK457796	Africa
MK457800	Africa
MK457813	Africa
MK457825	Africa
MK457949	Africa
MZ766718	Africa
MK457815	Africa
MZ766708	Africa
MZ766709	Africa
MZ766714	Africa
MZ766722	Africa
MZ766876	Africa
MZ766692	Africa

MZ766694	Africa
MZ766867	Africa
MK458073	Africa
MK458075	Africa
MK458078	Africa
MK458086	Africa
MK458079	Africa
MZ766680	Africa
MK458091	Africa
MK458089	Africa
AF290030	Africa
MZ766640	Africa
MZ766641	Africa
MK458123	Africa
MK458126	Africa
MZ766607	Africa
MZ766618	Africa
MZ766608	Africa
MZ766612	Africa
MZ766616	Africa
MZ766629	Africa
MZ766630	Africa
MZ766611	Africa
MZ766615	Africa
MZ766635	Africa
MZ766812	Africa
MZ766610	Africa
MZ766621	Africa

AF110970	Africa
MZ766700	Africa
MZ766707	Africa
MZ766702	Africa
MZ766701	Africa
MZ766705	Africa
AF443080	Africa
MZ766601	Africa
MZ766778	Africa
MK457782	Africa
MZ766828	Africa
AF443115	Africa
MZ766725	Africa
MZ766592	Africa
MZ766596	Africa
MZ766650	Africa
MZ766655	Africa
MZ766667	Africa
MZ766669	Africa
MZ766674	Africa
MZ766651	Africa
MZ766664	Africa
MZ766666	Africa
MZ766840	Africa
MZ766849	Africa
AF110981	Africa
MZ766586	Africa
MK458157	Africa

MK457773	Africa
MK457786	Africa
MK457957	Africa
MK457829	Africa
MK457832	Africa
MK457884	Africa
MK457933	Africa
MK457887	Africa
MZ766678	Africa
MK457836	Africa
MK457831	Africa
MK457894	Africa
MK457909	Africa
MK457921	Africa
MK457833	Africa
MK458040	Africa
MK458048	Africa
MK458055	Africa
MK458062	Africa
MZ766686	Africa
MZ766858	Africa
MZ766744	Africa
MZ766900	Africa
MK457968	Africa
MZ766688	Africa
MK457971	Africa
MK457970	Africa
MK458018	Africa

MK458020	Africa
MK458033	Africa
MZ766687	Africa
MK457972	Africa
MK457974	Africa
MK458140	Africa
MK458142	Africa
MK457982	Africa
MK458000	Africa
MK458026	Africa
MZ766737	Africa
MZ766749	Africa
MK457892	Africa
KU319535	Africa
AY713417	Africa
KU319545	Africa
KU319549	Africa
KU319530	Africa
KU319531	Africa
KU319536	Africa
MN791494	Africa
MN791504	Africa
MN791532	Africa
MN791521	Africa
MN791503	Africa
MN791516	Africa
MN791519	Africa
MN791526	Africa

MN791528	Africa
MN791524	Africa
KT022362	Africa
KY111988	Africa
KY111990	Africa
KY112006	Africa
KY112009	Africa
KY112583	Africa
KY112014	Africa
KY112253	Africa
KY112257	Africa
KY112377	Africa
KY112381	Africa
KY112366	Africa
KY112367	Africa
KY112372	Africa
KY112382	Africa
KY112386	Africa
KC156473	Africa
KC156475	Africa
KC156490	Africa
KC156482	Africa
KC156474	Africa
KC156481	Africa
KC156494	Africa
KC156492	Africa
KC156478	Africa
KC156489	Africa

KC156493	Africa
KC156486	Africa
KP109527	Africa
KC156117	Africa
KC156118	Africa
KF527061	Africa
KF527062	Africa
KF527172	Africa
KF527064	Africa
KF527070	Africa
KF527066	Africa
JQ779256	Africa
JQ779259	Africa
JQ779258	Africa
JQ779282	Africa
JQ779285	Africa
JQ779272	Africa
JQ779267	Africa
JQ779277	Africa
KC156217	Africa
KC156347	Africa
KC156354	Africa
KC156365	Africa
KC156345	Africa
KC156353	Africa
KC156359	Africa
KC156367	Africa
MT781859	Africa

MT781707	Africa
MT781698	Africa
MT781928	Africa
MT781831	Africa
MT781719	Africa
KY112202	Africa
KY112207	Africa
KY112215	Africa
KY112263	Africa
KY112284	Africa
KY112305	Africa
KY112304	Africa
KY112274	Africa
MT781757	Africa
MT781922	Africa
KP109524	Africa
MT781826	Africa
MT781840	Africa
KP109526	Africa
KC156211	Africa
KC156213	Africa
KC156422	Africa
KC156405	Africa
KC156419	Africa
KC156426	Africa
KC156407	Africa
JQ779101	Africa
JX973836	Africa

JX973821	Africa
JX973833	Africa
JQ779124	Africa
JX973842	Africa
JX973820	Africa
JQ779114	Africa
JX973831	Africa
JX973829	Africa
JQ779116	Africa
JX973841	Africa
JX973838	Africa
JX973822	Africa
JQ779127	Africa
MT781821	Africa
MT781771	Africa
MT781697	Africa
MT781733	Africa
KC156300	Africa
KC156308	Africa
KC156310	Africa
KC156314	Africa
KC156304	Africa
KC156311	Africa
KC156309	Africa
KC156316	Africa
KC156313	Africa
MT781743	Africa
MT781850	Africa

MT781723	Africa
MT781675	Africa
MT781888	Africa
MT781808	Africa
MT781814	Africa
OK649289	Africa
OK649293	Africa
OK649294	Africa
OK649266	Africa
OK649270	Africa
OK649271	Africa
OK649274	Africa
OK649275	Africa
OK649276	Africa
OK649283	Africa
OK649290	Africa
MT942713	Africa
MT942714	Africa
MT942720	Africa
MT942867	Africa
MT942873	Africa
MT942874	Africa
MT942868	Africa
MZ642273	Africa
MW443189	Africa
MW443195	Africa
AY734551	Africa
AY253304	Africa

AY253313	Africa
AY253303	Africa
KX907356	Africa
AF286235	Africa
AF361874	Africa
AY253317	Africa
OM825657	Africa
OM825663	Africa
OM825664	Africa
OM825576	Africa
OM825580	Africa
OM825577	Africa
AY734559	Africa
KX907391	Africa
MN791903	Africa
MN791909	Africa
MN791911	Africa
MN791924	Africa
MN791916	Africa
MN791927	Africa
MN791929	Africa
JQ779148	Africa
JQ779153	Africa
JQ779165	Africa
JQ779159	Africa
JQ779156	Africa
JQ779167	Africa
JQ779158	Africa

JQ779163	Africa
JQ779151	Africa
MN791938	Africa
MN791962	Africa
MN791959	Africa
MN791950	Africa
MN791944	Africa
MN791945	Africa
OM825687	Africa
OM825690	Africa
OM825693	Africa
OM825695	Africa
OM825698	Africa
OM825702	Africa
OM825703	Africa
MN650550	Africa
MN650390	Africa
MN650469	Africa
KJ948657	Africa
DQ369994	Africa
AY043173	Africa
KU168308	Africa
DQ164110	Africa
JX976710	Africa
JX976720	Africa
JX976716	Africa
JX976730	Africa
JX976718	Africa

JX976729	Africa
JX976717	Africa
DQ056406	Africa
AY043174	Africa
DQ093597	Africa
DQ275647	Africa
DQ369989	Africa
DQ445635	Africa
EU293447	Africa
AY158534	Africa
DQ056415	Africa
DQ164109	Africa
DQ396378	Africa
DQ396376	Africa
DQ369990	Africa
DQ351220	Africa
MN097655	Africa
MN097656	Africa
DQ351218	Africa
AY463221	Africa
AY878070	Africa
KC156125	Africa
DQ056412	Africa
DQ275658	Africa
DQ093604	Africa
EU293449	Africa
DQ396375	Africa
MN611462	Africa

MN611463	Africa
MN611465	Africa
AY878065	Africa
AY901970	Africa
DQ396364	Africa
KT183281	Africa
KT183283	Africa
AY463226	Africa
KT183190	Africa
KT183193	Africa
MK643581	Africa
MK643677	Africa
AY901978	Africa
DQ351233	Africa
DQ396394	Africa
GQ999988	Africa
DQ369988	Africa
DQ369978	Africa
DQ275642	Africa
AY162224	Africa
DQ396365	Africa
DQ011177	Africa
DQ351237	Africa
JX976670	Africa
JX976677	Africa
JX976671	Africa
JX976689	Africa
JX976672	Africa

JX976693	Africa
AY878061	Africa
DQ396389	Africa
KT183303	Africa
KT183305	Africa
KT183310	Africa
KC156124	Africa
KC156127	Africa
KC156128	Africa
KT183254	Africa
KT183255	Africa
KT183136	Africa
KT183138	Africa
AY901979	Africa
DQ011174	Africa
JN687639	Africa
MK643588	Africa
MK643604	Africa
MK643610	Africa
MK643614	Africa
MK643687	Africa
MK643690	Africa
MK643756	Africa
MK643776	Africa
MK643792	Africa
MK643797	Africa
MK643615	Africa
MK643743	Africa

MK643744	Africa
MK643746	Africa
MK643779	Africa
MK643802	Africa
ON862683	Africa
ON862692	Africa
MK643631	Africa
MK643698	Africa
GQ999987	Africa
AY463223	Africa
MN097669	Africa
MN097671	Africa
MN097672	Africa
JN687643	Africa
JN687645	Africa
MN097564	Africa
MN097575	Africa
MN097580	Africa
MN097579	Africa
MN097566	Africa
DQ275653	Africa
KT183105	Africa
KT183142	Africa
GQ999985	Africa
AY901981	Africa
DQ445633	Africa
KT183084	Africa
MN611468	Africa

DQ011180	Africa
JN687619	Africa
JN687620	Africa
JX140664	Africa
KT183131	Africa
KT183133	Africa
KT183186	Africa
KT183268	Africa
KT183270	Africa
KT183269	Africa
KT183177	Africa
AY878068	Africa
KT183151	Africa
KT183152	Africa
KT183317	Africa
KT183323	Africa
KT183325	Africa
KT183326	Africa
KT183123	Africa
KT183124	Africa
KT183127	Africa
DQ011178	Africa
DQ056409	Africa
DQ369992	Africa
KT183218	Africa
KT183219	Africa
DQ164118	Africa
JN687651	Africa

AY901975	Africa
MN097680	Africa
MN097681	Africa
MN097689	Africa
KT183196	Africa
KT183198	Africa
KT183199	Africa
DQ396397	Africa
AY703911	Africa
JQ779206	Africa
JQ779207	Africa
JQ779217	Africa
JQ779218	Africa
JQ779219	Africa
JQ779220	Africa
KT183227	Africa
KT183232	Africa
KT183233	Africa
KT183235	Africa
KT183234	Africa
MK643548	Africa
MK643549	Africa
DQ093602	Africa
KT183207	Africa
DQ396372	Africa
KT183261	Africa
JN687628	Africa
KT183089	Africa

KT183090	Africa
KT183099	Africa
KT183102	Africa
AY878064	Africa
DQ164116	Africa
AY585267	Africa
DQ396399	Africa
MN097648	Africa
KT183155	Africa
KT183156	Africa
KT183157	Africa
KT183161	Africa
KT183160	Africa
KT183208	Africa
KT183271	Africa
DQ351234	Africa
KY112077	Africa
KY112080	Africa
KY112078	Africa
KY112091	Africa
KY112083	Africa
KY112092	Africa
DQ396386	Africa
AY878060	Africa
DQ056413	Africa
MN202471	Africa
MH933714	Africa
ON862679	Africa

ON862682	Africa
KT183213	Africa
KT183215	Africa
MN097584	Africa
MN097600	Africa
MN097585	Africa
MN097588	Africa
MN097599	Africa
MN097590	Africa
MN097598	Africa
MN097596	Africa
EU293450	Africa
AY703910	Africa
KU749417	Africa
MN703148	Africa
KT183065	Africa
DQ396396	Africa
MN097603	Africa
MN097605	Africa
MN097634	Africa
MN097611	Africa
MN097633	Africa
MN097646	Africa
MN097638	Africa
MN097620	Africa
MN097610	Africa
MN097619	Africa
MN097635	Africa

EU293445	Africa
JN687629	Africa
JN687635	Africa
JN687636	Africa
KT183338	Africa
KP109516	Africa
MK643654	Africa
MK643661	Africa
MN703145	Africa
KT183168	Africa
KT183170	Africa
MN097554	Africa
MN097560	Africa
MN097562	Africa
DQ369980	Africa
JN687647	Africa
KT183240	Africa
KT183243	Africa
MN097661	Africa
MN097664	Africa
JX976634	Africa
JX976635	Africa
JX976652	Africa
JX976649	Africa
JX976639	Africa
JX976664	Africa
JX976666	Africa
KT183292	Africa

KT183295	Africa
KT183149	Africa
KM050298	Africa
AB254143	Africa
AB254144	Africa
AB254151	Africa
AB254152	Africa
KM050460	Africa
AB485646	Africa
AB485647	Africa
KM050076	Africa
KM050000	Africa
KM050474	Africa
KM050477	Africa
KM050085	Africa
KM050002	Africa
KM050270	Africa
KM049947	Africa
KM050362	Africa
KM050363	Africa
KM050089	Africa
KM049922	Africa
KM050636	Africa
KM050374	Africa
KM050376	Africa
KM050377	Africa
KM050008	Africa
KM049926	Africa

KM050004	Africa
KM049902	Africa
KM050691	Africa
KM050695	Africa
KM050696	Africa
KM050697	Africa
KM050698	Africa
KM050741	Africa
KM050335	Africa
KM050338	Africa
KM050339	Africa
KM050322	Africa
KM050325	Africa
KM050623	Africa
KM050689	Africa
MT194746	Africa
KM050144	Africa
KM050145	Africa
KM050146	Africa
KM050147	Africa
KM050631	Africa
KM050279	Africa
KM050280	Africa
KM050283	Africa
KM050069	Africa
KM050727	Africa
KM050016	Africa
MT194601	Africa

MT194606	Africa
MT194609	Africa
MT194605	Africa
KM050353	Africa
KM050354	Africa
KM050356	Africa
KM050357	Africa
KM050556	Africa
KM050026	Africa
KM050360	Africa
KM050361	Africa
KM050382	Africa
KM049996	Africa
KM049998	Africa
KM050179	Africa
KM050244	Africa
KM050326	Africa
KM050685	Africa
KM050705	Africa
KM050751	Africa
KM050182	Africa
KM050150	Africa
KM049919	Africa
KM049999	Africa
KM050122	Africa
KM050048	Africa
KM050050	Africa
KM050117	Africa

KM050087	Africa
KM049901	Africa
FJ496195	Africa
FJ496196	Africa
FJ496203	Africa
KM050457	Africa
KM050459	Africa
KM050458	Africa
KM050575	Africa
KM050648	Africa
KM050468	Africa
KM050469	Africa
KM050472	Africa
KM050227	Africa
KM050130	Africa
MT194483	Africa
MT194484	Africa
MT194491	Africa
KM050681	Africa
KM050080	Africa
AB254155	Africa
MT195233	Africa
KM050300	Africa
KM050301	Africa
KM050249	Africa
KM050701	Africa
KM050133	Africa
KM050449	Africa

KM050453	Africa
KM050454	Africa
KM050019	Africa
KM050632	Africa
KR820296	Africa
KR820298	Africa
KR820312	Africa
KM050633	Africa
KR820320	Africa
KR820319	Africa
KR820316	Africa
KR820311	Africa
KR820313	Africa
KM050358	Africa
KM050758	Africa
KR820327	Africa
KR820334	Africa
KR820335	Africa
KR820337	Africa
KR820339	Africa
KM049962	Africa
MT194175	Africa
MT194177	Africa
MT194138	Africa
KM050527	Africa
KM050529	Africa
KM050233	Africa
KM050236	Africa

KM050234	Africa
MT194284	Africa
MT194289	Africa
MT194286	Africa
KM050617	Africa
KM050219	Africa
KM050221	Africa
KM050073	Africa
MT194854	Africa
MT194855	Africa
MT194858	Africa
KM050196	Africa
KM050568	Africa
KM050210	Africa
KM050213	Africa
KM050214	Africa
KM050211	Africa
KM050641	Africa
KM050263	Africa
MT194988	Africa
KM050412	Africa
KM050639	Africa
KM050401	Africa
KM050403	Africa
KM050405	Africa
KM050515	Africa
KM050516	Africa
KM050098	Africa

KM050099	Africa
KM050723	Africa
KM050304	Africa
KM050305	Africa
KM049938	Africa
KM049940	Africa
KM050041	Africa
KM050044	Africa
KM050045	Africa
KM050329	Africa
KM050332	Africa
KM050333	Africa
KM050330	Africa
KM049921	Africa
KM050225	Africa
KM050061	Africa
KM050064	Africa
KM050066	Africa
KM050024	Africa
KM050341	Africa
KM050345	Africa
KM050380	Africa
KR820396	Africa
KR820416	Africa
KR820418	Africa
KR820399	Africa
KR820401	Africa
KR820404	Africa

KR820407	Africa
KR820414	Africa
KR820412	Africa
KM050137	Africa
KM050139	Africa
KM050141	Africa
KM050142	Africa
MT195136	Africa
MT195141	Africa
MT195495	Africa
MT195504	Africa
MT195513	Africa
MT195500	Africa
KM050767	Africa
KP109496	Africa
KR820447	Africa
KR820422	Africa
KR820436	Africa
KR820424	Africa
KR820429	Africa
KR820438	Africa
AB254148	Africa
KM050007	Africa
KM050286	Africa
KM050288	Africa
KM050287	Africa
KM050373	Africa
KM050317	Africa

KM050647	Africa
KM049965	Africa
KM049967	Africa
KM049970	Africa
KM050525	Africa
KM050729	Africa
KM050734	Africa
MT195390	Africa
MT195395	Africa
MT195402	Africa
MT195400	Africa
KM050596	Africa
KM050597	Africa
KM050598	Africa
KM050600	Africa
KM050669	Africa
KM050510	Africa
KM050511	Africa
KM050606	Africa
KM049973	Africa
KM049978	Africa
KM049976	Africa
KM050563	Africa
KM050656	Africa
KM050752	Africa
KM050495	Africa
KM050496	Africa
KM050389	Africa

KM050391	Africa
KM050392	Africa
KM050390	Africa
KM050131	Africa
KR820345	Africa
KR820352	Africa
KR820346	Africa
KR820355	Africa
KR820364	Africa
KM049914	Africa
KM049918	Africa
KM049991	Africa
FJ496209	Africa
KM050668	Africa
KM050254	Africa
KM050255	Africa
KM050709	Africa
MT347681	Africa
KM050503	Africa
KM050504	Africa
KM050505	Africa
KM050506	Africa
KM050507	Africa
KM049909	Africa
MT195151	Africa
FJ496190	Africa
FJ496194	Africa
KM050124	Africa

KM050592	Africa
KM050594	Africa
KM050106	Africa
KM050119	Africa
KM050442	Africa
KM049954	Africa
KM050161	Africa
KM050166	Africa
KM050416	Africa
KM050420	Africa
MT194317	Africa
MT194319	Africa
MT194324	Africa
MT194326	Africa
MT194322	Africa
MT194321	Africa
MT194343	Africa
KR820369	Africa
KR820381	Africa
KR820388	Africa
KR820389	Africa
KR820390	Africa
KR820373	Africa
KM050508	Africa
KM050440	Africa
KM050748	Africa
KM050544	Africa
KM050545	Africa

KU749426	Africa
KM050713	Africa
KM050079	Africa
KM050274	Africa
KM050295	Africa
KM050432	Africa
KM050077	Africa
KM049944	Africa
KM049983	Africa
KM049985	Africa
KM049989	Africa
KM050169	Africa
KM050170	Africa
KM050173	Africa
KY658711	Africa
KY658710	Africa
KY658704	Africa
KR861002	Africa
KR861210	Africa
KR861112	Africa
KR861010	Africa
KR860852	Africa
KR861233	Africa
KR860897	Africa
KR861036	Africa
KR860822	Africa
KR861238	Africa
KR860839	Africa

KR861088	Africa
KR860857	Africa
KR861150	Africa
KR860892	Africa
KR860875	Africa
KR861043	Africa
KR860828	Africa
KR860968	Africa
KR861251	Africa
KR861157	Africa
KR861026	Africa
KR861223	Africa
KC852932	Africa
KR860905	Africa
MZ678338	Africa
JN634488	Africa
KR860998	Africa
KR861123	Africa
KR860662	Africa
KR860678	Africa
KR860746	Africa
KR860618	Africa
KR860610	Africa
KR860725	Africa
KR860718	Africa
MN178985	Africa
MN179233	Africa
FR666622	Africa

FR666613	Africa
KU319539	Africa
HM635518	Africa
KC156357	Africa
KC156369	Africa
KC156356	Africa
KC156360	Africa
KC156364	Africa
KC156350	Africa
KC156361	Africa
KC156359	Africa
KC156355	Africa
KC156368	Africa
KC156353	Africa
KC156346	Africa
KC156216	Africa
KC156345	Africa
KC156344	Africa
KC156366	Africa
KC156358	Africa
KC156351	Africa
KC156362	Africa
KC156367	Africa
KC156371	Africa
KC156352	Africa
KC156349	Africa
KC156217	Africa
KC156363	Africa

KC156347	Africa
KC156354	Africa
KC156348	Africa
KC156365	Africa
KY112384	Africa
KY112364	Africa
KC156255	Africa
KF527143	Africa
KF527166	Africa
KF527182	Africa
KF527172	Africa
KF527174	Africa
KF527178	Africa
KF527183	Africa
KF527175	Africa
KF527184	Africa
KF527176	Africa
KF527177	Africa
KF527173	Africa
KF527179	Africa
KY112584	Africa
KY112583	Africa
KY112580	Africa
KY112579	Africa
KY112581	Africa
KY112578	Africa
KY112577	Africa
KY112582	Africa

KF527193	Africa
KF527079	Africa
KF527060	Africa
KF527073	Africa
KF527071	Africa
KF527072	Africa
KF527062	Africa
KF527064	Africa
KF527070	Africa
KF527063	Africa
KF527080	Africa
KF527074	Africa
KF527061	Africa
KF527077	Africa
KF527065	Africa
KF527076	Africa
KF527075	Africa
KF527067	Africa
KF527190	Africa
KF527188	Africa
KY112008	Africa
KY112009	Africa
KY111994	Africa
KY111996	Africa
KY112001	Africa
KY111998	Africa
KY112006	Africa
KY112003	Africa

KY111989	Africa
KY111987	Africa
KY111992	Africa
MF594935	Africa
KF927355	Africa
FN599776	Africa
MH234642	Africa
MH234641	Africa
MH234640	Africa
KJ948661	Africa
FJ199548	Africa
FJ199557	Africa
FJ199551	Africa
FJ199574	Africa
FJ199552	Africa
FJ199546	Africa
FJ199562	Africa
FJ199595	Africa
FJ199597	Africa
FJ199584	Africa
FJ199535	Africa
FJ199532	Africa
FJ199654	Africa
FJ199625	Africa
FJ199674	Africa
FJ199709	Africa
FJ199666	Africa
FJ199678	Africa

FJ199682	Africa
FJ199651	Africa
FJ199608	Africa
FJ199640	Africa
FJ199738	Africa
FJ199767	Africa
FJ199746	Africa
FJ199762	Africa
FJ199723	Africa
FJ199735	Africa
FJ199750	Africa
FJ199730	Africa
FJ199759	Africa
FJ199758	Africa
KT737121	Africa
KT736984	Africa
KT737054	Africa
KT736980	Africa
KT737137	Africa
KT737141	Africa
GU253419	Africa
GU253405	Africa
KT737002	Africa
KX228799	Africa
MN703147	Africa
JN188292	Africa
FJ647145	Africa
KU168308	Africa

KY099043	Africa
KY099068	Africa
KY099222	Africa
GQ999975	Africa
GQ999985	Africa
KT183085	Africa
KT183088	Africa
KT183087	Africa
KF906058	Africa
MW190033	Africa
MW190032	Africa
MW190035	Africa
MW190031	Africa
MW190034	Africa
MW190030	Africa
MW190036	Africa
MW189906	Africa
KT183203	Africa
KT183202	Africa
KT183201	Africa
KT183207	Africa
KT183206	Africa
KT183204	Africa
KT183245	Africa
KT183248	Africa
KT183246	Africa
KT183247	Africa
KT183249	Africa

KT183056	Africa
KT183061	Africa
KT183182	Africa
KT183179	Africa
KT183187	Africa
KT183180	Africa
KT183178	Africa
KT183185	Africa
KT183181	Africa
KT183184	Africa
JN664998	Africa
KX902349	Africa
KU253082	Africa
KT893169	Africa
MN611466	Africa
MN611468	Africa
MN611467	Africa
MN097577	Africa
MN097567	Africa
MN097658	Africa
MN097654	Africa
MN097657	Africa
JN176306	Africa
DQ093597	Africa
DQ396384	Africa
DQ445634	Africa
DQ396377	Africa
DQ093607	Africa

DQ369990	Africa
DQ396378	Africa
AY901967	Africa
AY901969	Africa
DQ164106	Africa
DQ056411	Africa
DQ164107	Africa
DQ396393	Africa
DQ164126	Africa
DQ011179	Africa
DQ164112	Africa
DQ164110	Africa
DQ056417	Africa
DQ369994	Africa
MH709732	Africa
MH709397	Africa
KF530913	Africa
KF530910	Africa
KF530924	Africa
KF530918	Africa
KF530920	Africa
KF530915	Africa
KX907424	Africa
KX907379	Africa
KX907337	Africa
KX907398	Africa
KX907390	Africa
GQ427125	Africa

GQ427124	Africa
GQ427130	Africa
GQ427128	Africa
GQ427126	Africa
GQ433841	Africa
GQ433860	Africa
GQ433828	Africa
KC018873	Africa
KC018874	Africa
HM119879	Africa
HM120051	Africa
HM119838	Africa
KM050740	Africa
KM050741	Africa
KM050649	Africa
KM050650	Africa
KM050118	Africa
KM050646	Africa
KM050088	Africa
KM050089	Africa
KM050645	Africa
KM050105	Africa
KM050145	Africa
KM050144	Africa
KM050146	Africa
KM050147	Africa
KM050320	Africa
KM050240	Africa

KM050267	Africa
KM050266	Africa
KM050268	Africa
KM050460	Africa
KM050461	Africa
KM050353	Africa
KM050352	Africa
KM050264	Africa
KM050265	Africa
KM050375	Africa
KM050269	Africa
KM050374	Africa
KM050354	Africa
KM050538	Africa
KM050474	Africa
KM050395	Africa
KM050394	Africa
KM050270	Africa
KM050376	Africa
KM050355	Africa
KM050527	Africa
KM050514	Africa
KM050515	Africa
KM050340	Africa
KM050476	Africa
KM050396	Africa
KM050377	Africa
KM050271	Africa

KM050356	Africa
KM050528	Africa
KM050494	Africa
KM050477	Africa
KM050397	Africa
KM050378	Africa
KM050357	Africa
KM050529	Africa
KM050559	Africa
KM050478	Africa
KM050565	Africa
KM050564	Africa
KM050379	Africa
KM050398	Africa
KM049947	Africa
KM050623	Africa
KM050076	Africa
KM049903	Africa
KM050685	Africa
KM050090	Africa
KM050745	Africa
KM050746	Africa
KM050530	Africa
KM050085	Africa
KM049902	Africa
KM050086	Africa
KM049923	Africa
KM050002	Africa

KM050069	Africa
KM050751	Africa
KM049922	Africa
KM050004	Africa
KM050561	Africa
KM050087	Africa
KM050021	Africa
KM050080	Africa
KM050008	Africa
KM050727	Africa
KM049919	Africa
KM050479	Africa
KM050722	Africa
KM050651	Africa
KM050706	Africa
KM050635	Africa
KM050636	Africa
KM050637	Africa
KM050681	Africa
KM050589	Africa
KM050588	Africa
KM050631	Africa
KM050717	Africa
KM050643	Africa
KM050703	Africa
KM050726	Africa
KM050719	Africa
KM050113	Africa

KM050115	Africa
KM050399	Africa
KM050739	Africa
KM050112	Africa
KM050179	Africa
KM050531	Africa
KM050125	Africa
KM050055	Africa
KM050130	Africa
KM050132	Africa
KM050231	Africa
KM050034	Africa
KM050000	Africa
KM050592	Africa
KM050035	Africa
KM049935	Africa
KM050653	Africa
KM050673	Africa
KM050691	Africa
KM050037	Africa
JN132227	Africa
MT194492	Africa
MT194491	Africa
MT194488	Africa
KM050010	Africa
MT194494	Africa
MT194490	Africa
MT194489	Africa

KM050091	Africa
KM050009	Africa
MT194497	Africa
MT194498	Africa
MT347680	Africa
KM050054	Africa
MZ662681	Americas
MZ662690	Americas
MZ662593	Americas
MZ662596	Americas
MZ662600	Americas
MZ662649	Americas
MZ662620	Americas
ON816268	Americas
ON816278	Americas
ON816287	Americas
ON816272	Americas
ON816672	Americas
ON816826	Americas
ON816945	Americas
ON816801	Americas
ON816961	Americas
ON816685	Americas
ON816822	Americas
MW881719	Americas
ON816571	Americas
ON816051	Americas
ON816069	Americas

ON816059	Americas
ON817063	Americas
MZ662753	Americas
ON816178	Americas
ON816194	Americas
ON816160	Americas
ON816167	Americas
ON816185	Americas
AY779555	Americas
ON816458	Americas
ON816462	Americas
ON816472	Americas
ON816475	Americas
ON816480	Americas
ON816459	Americas
ON816489	Americas
ON816499	Americas
ON816473	Americas
ON816481	Americas
ON816365	Americas
ON816377	Americas
ON816378	Americas
ON816391	Americas
JF320428	Americas
MZ662695	Americas
ON816355	Americas
KR914678	Americas
EU839597	Americas

MT794801	Americas
MT794804	Americas
HM030565	Americas
MZ922679	Americas
DQ487188	Americas
DQ487190	Americas
MK383432	Americas
MK384653	Americas
MK384840	Americas
MK383497	Americas
MK383524	Americas
MK383539	Americas
MK383542	Americas
MW754077	Americas
MW754090	Americas
MZ080807	Americas
MZ080813	Americas
FJ469683	Americas
OQ092465	Americas
MW059278	Americas
MW059282	Americas
MW059352	Americas
MW059354	Americas
MW059376	Americas
MW059379	Americas
MW059283	Americas
MW059305	Americas
MW059321	Americas

MW059381	Americas
OP700958	Americas
KX505572	Americas
FJ469730	Americas
JF320338	Americas
MW063014	Americas
JF689872	Americas
JN599165	Americas
OM209961	Americas
AY331289	Americas
AY835772	Americas
AY835773	Americas
MK383394	Americas
MK383402	Americas
MK383418	Americas
MK384667	Americas
MK384055	Americas
MK383632	Americas
MK383690	Americas
MK384037	Americas
MK385071	Americas
MK383521	Americas
MK383577	Americas
MK384955	Americas
MK383590	Americas
MK383677	Americas
MK383629	Americas
MK384086	Americas

MK383623	Americas
MK384124	Americas
MK383835	Americas
MK383655	Americas
MK384559	Americas
FJ496147	Americas
FJ469691	Americas
KT124813	Americas
KU678034	Americas
KY778437	Americas
MH843890	Americas
MH843912	Americas
KY778439	Americas
MH843914	Americas
JN944922	Americas
JN944926	Americas
MG196864	Americas
FJ469738	Americas
KY766175	Americas
MW753873	Americas
MH843747	Americas
MH843766	Americas
MH843769	Americas
MW753734	Americas
MW753810	Americas
MW753877	Americas
OM208914	Americas
OM209011	Americas

JN024302	Americas
JN024330	Americas
OM205595	Americas
MZ922551	Americas
ON015295	Americas
JN024564	Americas
KU678071	Americas
OL872906	Americas
KT284378	Americas
MW059455	Americas
MW059473	Americas
OM203922	Americas
OM203929	Americas
OM203961	Americas
OM203971	Americas
OM203979	Americas
OM204014	Americas
OM204038	Americas
OM204179	Americas
OM204215	Americas
OM204228	Americas
OM204236	Americas
OM204339	Americas
OM203966	Americas
MW059511	Americas
OM209448	Americas
OM209455	Americas
MW059484	Americas

OM204043	Americas
OM204169	Americas
KF526265	Americas
ON015115	Americas
ON015123	Americas
ON015155	Americas
ON015152	Americas
ON015145	Americas
ON015130	Americas
ON015163	Americas
FJ495823	Americas
FJ495826	Americas
FJ496089	Americas
KY112066	Americas
FJ469754	Americas
KU677989	Americas
KU677993	Americas
KU677997	Americas
KU678017	Americas
MG196885	Americas
MG196886	Americas
MG196888	Americas
OM205955	Americas
JQ403072	Americas
DQ853444	Americas
MG196848	Americas
MH843820	Americas
MW753935	Americas

MW753917	Americas
KU678162	Americas
ON015219	Americas
ON015260	Americas
ON015220	Americas
ON015233	Americas
JF320181	Americas
JF320200	Americas
JN024207	Americas
JN024211	Americas
JN024212	Americas
JN024221	Americas
JN024260	Americas
JN024269	Americas
JN024229	Americas
JN024228	Americas
MZ922526	Americas
MZ922731	Americas
MG197115	Americas
JF320263	Americas
JF320161	Americas
KY112485	Americas
KY112491	Americas
KY112530	Americas
MK115484	Americas
MK115551	Americas
MK115561	Americas
MK115574	Americas

MT191041	Americas
MK115535	Americas
MK115587	Americas
MT191059	Americas
MT190020	Americas
MZ922802	Americas
OL873079	Americas
OM204996	Americas
OM205004	Americas
JF320549	Americas
MG197017	Americas
MN691961	Americas
JF320628	Americas
MW062894	Americas
KY112130	Americas
KY112430	Americas
KY112444	Americas
KY112433	Americas
KY112443	Americas
FJ495941	Americas
FJ495942	Americas
KC312513	Americas
KC312529	Americas
KC312536	Americas
MN466977	Americas
MN467034	Americas
MG196838	Americas
MT998621	Americas

MT998625	Americas
MT998628	Americas
MT998636	Americas
MT998663	Americas
MT998680	Americas
MK169449	Americas
MK169467	Americas
MK169488	Americas
MK383490	Americas
MK383540	Americas
MK383562	Americas
MK384132	Americas
MK384224	Americas
MK385438	Americas
JF320113	Americas
JN024443	Americas
JN024444	Americas
JN024507	Americas
JN024471	Americas
JN024401	Americas
JN024408	Americas
JN024407	Americas
MG196914	Americas
OM206194	Americas
JF320326	Americas
OM206917	Americas
OM209677	Americas
JN024101	Americas

JN024146	Americas
JN024148	Americas
JN024152	Americas
JN024572	Americas
JN024161	Americas
JN024187	Americas
JN024177	Americas
FJ469731	Americas
MT189316	Americas
MT190815	Americas
MT190819	Americas
MT190820	Americas
MT190828	Americas
MT190840	Americas
MT190842	Americas
MT190862	Americas
MT190885	Americas
MT190888	Americas
MT190978	Americas
MT190986	Americas
OM209372	Americas
OM209418	Americas
OM209423	Americas
OM209427	Americas
MT190781	Americas
MW059264	Americas
MT190989	Americas
MT191004	Americas

OM203886	Americas
OM207248	Americas
MK169683	Americas
MK169758	Americas
MK169710	Americas
MK169812	Americas
MW062436	Americas
MZ922982	Americas
JF320608	Americas
JF320612	Americas
MW062646	Americas
MG196677	Americas
MG196711	Americas
MW754325	Americas
MW754474	Americas
MZ080664	Americas
MZ080676	Americas
MZ080684	Americas
MZ080696	Americas
FJ496077	Americas
JF320185	Americas
JF320194	Americas
OM207969	Americas
OM208065	Americas
OM208086	Americas
OM208097	Americas
OM208122	Americas
JF320280	Americas

MW062274	Americas
MW062316	Americas
OM207374	Americas
OM209714	Americas
MW062382	Americas
MK115415	Americas
MK115475	Americas
MK115429	Americas
MG196745	Americas
MG196749	Americas
MG196752	Americas
JF320404	Americas
KC312414	Americas
KC312419	Americas
KC473833	Americas
KU749387	Americas
EU547186	Americas
MG197195	Americas
MK169546	Americas
MK169622	Americas
MK169550	Americas
MK169598	Americas
MK169610	Americas
MK169636	Americas
MK169642	Americas
MK169655	Americas
MG197182	Americas
OM207540	Americas

OM207670	Americas
OM207672	Americas
OM207694	Americas
OM209719	Americas
OM209721	Americas
OM209738	Americas
OM209744	Americas
OM209755	Americas
OM209791	Americas
OM209792	Americas
OM209806	Americas
OM209813	Americas
OM209820	Americas
OM209833	Americas
OM209837	Americas
OM209867	Americas
OM209881	Americas
OM209886	Americas
OM209899	Americas
OM209920	Americas
OM209921	Americas
OM209937	Americas
OM209891	Americas
OM207696	Americas
OM207565	Americas
MW062692	Americas
MW063094	Americas
MW059624	Americas

MW059643	Americas
OM204456	Americas
MW063009	Americas
OM204569	Americas
OM204583	Americas
OM204605	Americas
MG196773	Americas
MK114658	Americas
MT191073	Americas
MT191085	Americas
MK114704	Americas
MK114703	Americas
MK115138	Americas
MW061272	Americas
MK115184	Americas
MK115272	Americas
MK115328	Americas
MK115335	Americas
MW061106	Americas
MW061253	Americas
MN090420	Americas
MK115217	Americas
MN090429	Americas
MN090390	Americas
MK115179	Americas
MN090417	Americas
MK115259	Americas
MW061157	Americas

MW061309	Americas
MK115302	Americas
MK115198	Americas
MN090399	Americas
MW061141	Americas
MW061146	Americas
MK115159	Americas
OM206323	Americas
OM206340	Americas
OM206347	Americas
OM206398	Americas
OM206466	Americas
OM206476	Americas
OM206508	Americas
OM206518	Americas
OM206558	Americas
OM206597	Americas
OM206620	Americas
OM206647	Americas
OM206662	Americas
OM206701	Americas
OM206715	Americas
OM206746	Americas
OM206760	Americas
OM206358	Americas
MW062969	Americas
KC312542	Americas
KC312563	Americas

KC312566	Americas
OM207134	Americas
MW924802	Americas
MN467313	Americas
MN467318	Americas
MN467340	Americas
MN467336	Americas
OM207516	Americas
OM207517	Americas
OM207520	Americas
MT189928	Americas
MT189946	Americas
MT189974	Americas
MT189984	Americas
MW060186	Americas
MG197200	Americas
ON015273	Americas
JN687667	Americas
JN687668	Americas
JN944898	Americas
MK383386	Americas
MK383501	Americas
MK384200	Americas
MK383449	Americas
MK384347	Americas
JQ403062	Americas
OM208362	Americas
OM208557	Americas

OM208647	Americas
OM208472	Americas
OM208595	Americas
OM208657	Americas
OM208473	Americas
MG197212	Americas
MG197214	Americas
MK114991	Americas
MK115069	Americas
MN090353	Americas
MW061036	Americas
MK115105	Americas
MW061064	Americas
MK115019	Americas
MK115056	Americas
JF320126	Americas
OP701040	Americas
FJ469740	Americas
OM206023	Americas
OM206040	Americas
OM206069	Americas
OM209519	Americas
OM209530	Americas
OM209545	Americas
OM209563	Americas
OM209533	Americas
MG196690	Americas
MG196988	Americas

MG196926	Americas
KF526323	Americas
MK115709	Americas
MK115721	Americas
MK115723	Americas
MK115853	Americas
MN090523	Americas
MN090544	Americas
MN090563	Americas
MN090564	Americas
MN090607	Americas
MN090609	Americas
MN090631	Americas
MN090632	Americas
MW061384	Americas
MW061412	Americas
MW061430	Americas
MW061468	Americas
MW061470	Americas
MW061487	Americas
MW061496	Americas
MW061507	Americas
MW061538	Americas
MW061573	Americas
MW061583	Americas
MW061598	Americas
MW061630	Americas
MW061761	Americas

MK115724	Americas
MN090530	Americas
MN090620	Americas
MN090705	Americas
MN090706	Americas
MW061587	Americas
MW061617	Americas
MW061620	Americas
MW061685	Americas
MW061749	Americas
MK115743	Americas
MK115777	Americas
MK115850	Americas
MN090670	Americas
MW061527	Americas
MN090662	Americas
MW061508	Americas
MN090652	Americas
MN090555	Americas
MN090674	Americas
MN090672	Americas
OM203676	Americas
MG196780	Americas
JN687690	Americas
KY778615	Americas
FJ469718	Americas
JF320444	Americas
JF320448	Americas

MT191013	Americas
MW060050	Americas
MW060016	Americas
MG197035	Americas
MW753264	Americas
MZ080906	Americas
MG196979	Americas
JQ403036	Americas
MG197081	Americas
MG197143	Americas
JN944909	Americas
MZ922501	Americas
MG197025	Americas
MK169506	Americas
MK169532	Americas
MG197133	Americas
JN397365	Americas
OM204773	Americas
MK114869	Americas
MW060647	Americas
MW060403	Americas
MN090213	Americas
MK114947	Americas
MW063032	Americas
MW060367	Americas
MN090329	Americas
MW060541	Americas
MW060625	Americas

MK114926	Americas
MK114960	Americas
MW063046	Americas
MN090231	Americas
MW060274	Americas
MW060381	Americas
MW060314	Americas
MW060623	Americas
MN090256	Americas
MK114889	Americas
OM209651	Americas
KC473828	Americas
MK169860	Americas
MK169876	Americas
MG197050	Americas
KY766153	Americas
MW754582	Americas
MZ922700	Americas
MZ922703	Americas
OM209013	Americas
OM208819	Americas
MT190764	Americas
MT191178	Americas
MT191206	Americas
MT191221	Americas
MT191222	Americas
MT191224	Americas
MT191188	Americas

MT191207	Americas
MW062194	Americas
MW062210	Americas
OM205938	Americas
KY778459	Americas
KY778473	Americas
KY778487	Americas
KY778548	Americas
MW754161	Americas
MW754164	Americas
MW754262	Americas
MG196952	Americas
MG196962	Americas
MW062429	Americas
MW062453	Americas
MW062478	Americas
MW062538	Americas
OM206141	Americas
KY778295	Americas
MW753958	Americas
MW062571	Americas
MW062677	Americas
MW062801	Americas
MW062824	Americas
MK385425	Americas
MZ922726	Americas
OM207901	Americas
MW754315	Americas

MW754349	Americas
MZ080628	Americas
MZ080711	Americas
MW062367	Americas
OM207357	Americas
MW060938	Americas
MW060981	Americas
KT982201	Americas
KF526214	Americas
KF526220	Americas
KF526219	Americas
KF526223	Americas
MW262772	Americas
MW262774	Americas
ON816123	Americas
ON816077	Americas
ON816749	Americas
MZ662575	Americas
MZ662669	Americas
MZ662673	Americas
MZ662679	Americas
MZ662712	Americas
KJ704795	Americas
KJ704794	Americas
KJ704791	Americas
KJ704792	Americas
KJ704789	Americas
KJ704793	Americas

EF363124	Americas
JF689859	Americas
MW754704	Americas
MW754583	Americas
MW754564	Americas
MZ922624	Americas
MZ922518	Americas
MZ922701	Americas
MZ922696	Americas
MZ922614	Americas
MZ922656	Americas
MZ922662	Americas
MW754690	Americas
MW754534	Americas
MW754524	Americas
MW754549	Americas
MW754535	Americas
MW754520	Americas
MN919177	Americas
FJ469736	Americas
MN692148	Americas
ON015197	Americas
MN692074	Americas
MN691959	Americas
DQ383748	Americas
MH078531	Americas
MK041556	Americas
FJ195088	Americas

KT427777	Americas
KJ849767	Americas
KT427716	Americas
KT427727	Americas
KT427770	Americas
KT427870	Americas
MG571982	Americas
KT427785	Americas
KT427675	Americas
KT427826	Americas
MK041562	Americas
KT427676	Americas
KT427724	Americas
KT427709	Americas
JN692467	Americas
JN692434	Americas
KX228818	Americas
MK041564	Americas
KT427810	Americas
KT427678	Americas
KT427806	Americas
MK041561	Americas
KT427674	Americas
JF320240	Americas
MH078554	Americas
JF320027	Americas
AY781126	Americas
FJ659743	Americas

GU807516	Americas
AB750367	Asia
GU647197	Asia
JF932471	Asia
KT191962	Asia
KP178432	Asia
KT191994	Asia
JF932473	Asia
JQ901049	Asia
JQ901062	Asia
KC596066	Asia
JQ901055	Asia
JQ898276	Asia
KC898995	Asia
KF250404	Asia
JQ898262	Asia
JQ898265	Asia
JQ898242	Asia
JQ898247	Asia
JQ898249	Asia
FJ388962	Asia
JF683742	Asia
FJ388933	Asia
JF683743	Asia
JF683750	Asia
JF683765	Asia
JF683775	Asia
FJ388927	Asia

JF683777	Asia
JF683803	Asia
JF683757	Asia
FJ388901	Asia
DQ207941	Asia
AY255823	Asia
AY255826	Asia
AB023804	Asia
AF067159	Asia
KP109486	Asia
KP109485	Asia
KC156186	Asia
KC156193	Asia
KC156206	Asia
KC156207	Asia
KC156209	Asia
KC156198	Asia
KC156200	Asia
KC156204	Asia
AF067154	Asia
AY049709	Asia
AY049710	Asia
AY049711	Asia
KY713231	Asia
KP109488	Asia
KY713236	Asia
KX069220	Asia
EF469243	Asia

KX069227	Asia
KY713229	Asia
KF766542	Asia
KY713228	Asia
AB428555	Asia
HQ026561	Asia
MH054720	Asia
HQ026588	Asia
MK577478	Asia
MN043598	Asia
JX149532	Asia
KF561442	Asia
MW660374	Asia
KJ140156	Asia
MW660525	Asia
HQ026618	Asia
KX692736	Asia
MT582421	Asia
DQ837381	Asia
MW405330	Asia
MW881608	Asia
MW405337	Asia
KX692426	Asia
KX692347	Asia
MT021899	Asia
KU341725	Asia
KU341728	Asia
MK493076	Asia

JX447788	Asia
JX447790	Asia
JX447163	Asia
JN248333	Asia
JN248354	Asia
MN792386	Asia
MN792395	Asia
JN860769	Asia
ON863202	Asia
KF250380	Asia
KT893516	Asia
KF835515	Asia
MK287325	Asia
MN908706	Asia
HG421498	Asia
MH757216	Asia
KP109488	Asia
KP109480	Asia
KT175207	Asia
KT175211	Asia
KT175215	Asia
KT175206	Asia
KT175214	Asia
FJ878957	Asia
EU683792	Asia
KC156184	Asia
KC156204	Asia
KC156200	Asia

KC156210	Asia
KC156192	Asia
KC156197	Asia
KC156187	Asia
KC156181	Asia
KC156201	Asia
KC156194	Asia
KC156196	Asia
KC156182	Asia
KC156190	Asia
KC156209	Asia
KC156188	Asia
KC156195	Asia
KU341724	Asia
JN023035	Asia
KX692617	Asia
MN043576	Asia
MN043577	Asia
KX692429	Asia
HQ026550	Asia
MK577481	Asia
HQ026563	Asia
HQ026548	Asia
MN043588	Asia
MN043596	Asia
MW660419	Asia
MN043587	Asia
HQ026591	Asia

HM210885	Asia
HQ026601	Asia
KJ140262	Asia
MK577478	Asia
HQ026560	Asia
KJ140250	Asia
MH054671	Asia
HQ026598	Asia
KJ140260	Asia
MH054877	Asia
MZ041424	Europe
MZ955771	Europe
MZ955775	Europe
MZ041473	Europe
MW881661	Europe
MW881739	Europe
MW881745	Europe
MW881727	Europe
OQ596941	Europe
OQ596888	Europe
OQ596896	Europe
OQ596913	Europe
MZ041513	Europe
MZ041568	Europe
MW881766	Europe
MW881767	Europe
MZ041307	Europe
MZ041279	Europe

MT417752	Europe
MN485979	Europe
OQ596925	Europe
OQ596935	Europe
OQ596927	Europe
MZ955733	Europe
MZ955740	Europe
MT417761	Europe
MZ041407	Europe
MH746264	Europe
KC797172	Europe
KC797178	Europe
KC797216	Europe
JQ403025	Europe
MK115622	Europe
MK115652	Europe
MK115633	Europe
MK115637	Europe
MK115691	Europe
MK115952	Europe
MK115963	Europe
MK115966	Europe
MK116019	Europe
MK116022	Europe
MK116024	Europe
MK116057	Europe
MK116072	Europe
MN090751	Europe

MN090767	Europe
MN090802	Europe
MK116047	Europe
MK115961	Europe
MK116088	Europe
MN090845	Europe
MK116055	Europe
KT124751	Europe
MK116112	Europe
MN090871	Europe
MK116122	Europe
MW062085	Europe
MN090854	Europe
MW062012	Europe
MN090910	Europe
MW062082	Europe
MW063069	Europe
MT190631	Europe
MW061920	Europe
MN090920	Europe
MW061998	Europe
EF514713	Europe
AJ006287	Europe
GQ386774	Europe
KT259311	Europe
GQ386781	Europe
OK181199	Europe
OK181180	Europe

KC473843	Europe
KT200351	Europe
KT276262	Europe
KU685585	Europe
FJ670525	Europe
GU362886	Europe
EU884500	Europe
JX140662	Europe
EU786681	Europe
JX140665	Europe
KY496624	Europe
KT276258	Europe
K03455	Europe
KJ925006	Europe
ON421463	Europe
OP994538	Europe
ON421509	Europe
OP994633	Europe
OP994705	Europe
OP994482	Europe
JX140652	Europe
KF716496	Europe
OP994353	Europe
OP994361	Europe
OP994362	Europe
ON421501	Europe
OP994354	Europe
MF109385	Europe

HM586208	Europe
MT794865	Europe
MT794871	Europe
MT794876	Europe
MT794908	Europe
HM586212	Europe
MT794197	Europe
MT794220	Europe
MF109479	Europe
MF109363	Europe
MT794042	Europe
MT794045	Europe
MT794085	Europe
MT794062	Europe
MF109499	Europe
MF109620	Europe
MF109596	Europe
MF109663	Europe
MF109552	Europe
MF109616	Europe
MH663809	Europe
MH663895	Europe
MH663886	Europe
MH663892	Europe
MH663894	Europe
MH663815	Europe
MH663864	Europe
MH663871	Europe

MH663872	Europe
MH663844	Europe
MH663900	Europe
MF109706	Europe
MF109403	Europe
MF109593	Europe
MF109599	Europe
MF109522	Europe
MF109695	Europe
MF109535	Europe
MF109539	Europe
MF109557	Europe
MF109412	Europe
MF373161	Europe
MF373126	Europe
KP411836	Europe
KP411832	Europe
MF373158	Europe
KP411831	Europe
MF373178	Europe
MF373165	Europe
MF373131	Europe
MF373187	Europe
MF373184	Europe
KP411834	Europe
MF373197	Europe
MF373170	Europe
MF373138	Europe

MF373132	Europe
DQ823362	Europe
ON421497	Europe
ON421494	Europe
ON421495	Europe
ON421493	Europe
ON421496	Europe
MK086129	Europe
JX140662	Europe
MN055643	Europe
MF373126	Europe
GU324885	Europe
GQ848145	Europe
KU498871	Europe
KU499084	Europe
KU499018	Europe
FJ653087	Europe
MF109593	Europe
MF109513	Europe
MF109535	Europe
MF109567	Europe
OK532665	Oceania
OK532720	Oceania
OK532721	Oceania
OK532680	Oceania
OK532704	Oceania
OK532706	Oceania
OK532716	Oceania

OK532734	Oceania
OK532757	Oceania
OK532764	Oceania
OK532802	Oceania
OK532807	Oceania
OK532798	Oceania
OK532917	Oceania
OK532972	Oceania
OK533000	Oceania
OK532942	Oceania
OK532454	Oceania
OK532457	Oceania
OK533189	Oceania
OK533224	Oceania
OK532484	Oceania
OK533178	Oceania
OK532500	Oceania
OK532490	Oceania
OK533276	Oceania
OK533177	Oceania
OK533194	Oceania
OK533277	Oceania
OK533263	Oceania
OK532471	Oceania
OK532505	Oceania
OK533259	Oceania
OK533219	Oceania
OK533261	Oceania

OP701120	Oceania
OK532389	Oceania
OP425427	Oceania
OP701276	Oceania
OP701312	Oceania
OK532837	Oceania
OK532844	Oceania
OK532882	Oceania
OK532897	Oceania
OK532865	Oceania
OK532899	Oceania
OK532907	Oceania
OK532874	Oceania
OK532648	Oceania
OK532650	Oceania
OK532651	Oceania
OK532655	Oceania
DQ676880	Oceania
DQ676882	Oceania
OP701541	Oceania
OP701357	Oceania
DQ676876	Oceania
OK532554	Oceania
OK533034	Oceania
OK533050	Oceania
OK533094	Oceania
OK533111	Oceania
OK533127	Oceania

OK533144	Oceania
OK532598	Oceania
OK532602	Oceania
OK533040	Oceania
OK533062	Oceania
OK533070	Oceania
OK533081	Oceania
OK533098	Oceania
OK533157	Oceania
OK533165	Oceania
OK533085	Oceania
OK533166	Oceania
OK533120	Oceania
OK533035	Oceania
OK533126	Oceania
AF042104	Oceania
OP701563	Oceania
OP701579	Oceania
OK532428	Oceania
